# Metabolic effects of 3-hydroxybutyrate infusion in individuals with type 1 diabetes compared with healthy control participants: a randomised crossover trial showing intact feedback suppression of lipolysis

**DOI:** 10.1007/s00125-025-06423-5

**Published:** 2025-04-10

**Authors:** Maj Bangshaab, Mads V. Svart, Nikolaj Rittig, Mette G. B. Pedersen, Jens Voigt, Niels Jessen, Niels Møller

**Affiliations:** 1 Medical/Steno, Aarhus Research Laboratory, Department of Clinical Medicine, Aarhus University, Aarhus, Denmark; 2 Steno Diabetes Center Aarhus, Aarhus University Hospital, Aarhus N, Denmark; 3 Department of Internal Medicine and Endocrinology, Aarhus University Hospital, Aarhus N, Denmark

**Keywords:** Adipose tissue signalling, 3-Hydroxybutyrate, Insulin resistance, Ketoacidosis, Ketones, Lipolysis, Metabolism, Type 1 diabetes

## Abstract

**Aims/hypothesis:**

Diabetic ketoacidosis remains a severe complication in type 1 diabetes, arising from insufficient insulin levels and accelerated lipolytic rate, leading to increased β-oxidation of NEFA and ketone body production in the liver. The ketone body 3-hydroxybutyrate (3-OHB) inhibits lipolysis in healthy individuals. The current study aimed to test whether this feedback suppression of lipolysis by 3-OHB is disrupted in individuals with type 1 diabetes.

**Methods:**

We used a single-blind, randomised, crossover design to study ten men diagnosed with type 1 diabetes and ten healthy control participants. Eligibility criteria were male sex, age ≥18 years, BMI of 19–26 kg/m^2^ and no severe comorbidities/diseases. Following an overnight fast, each participant received two 3 h i.v. infusions: (i) sodium-d/l-3-OHB and (ii) iso-osmolar saline (NaCl), separated by a 1 h washout period. The order of the two interventions was assigned by randomisation for each participant. Participants were blinded to the allocation throughout the study day, but investigators were aware of the assigned intervention order. We evaluated the lipolytic rate and glucose turnover using [9,10-^3^H]palmitate and [3-^3^H]glucose tracers. Additionally, adipose tissue signalling was quantified using western blotting techniques in subcutaneous abdominal adipose tissue biopsies. The primary endpoint measure was palmitate flux (lipolytic rate).

**Results:**

During the infusion of 3-OHB, the d/l-3-OHB blood concentrations increased to 3.3 ± 0.7 mmol/l in participants with type 1 diabetes compared with 2.9 ± 0.5 mmol/l in control participants (*p*=0.03). The infusion effectively suppressed the lipolytic rates by more than 50% (*p*<0.001) and reduced circulating NEFA by approximately 0.5 mmol/l (*p*<0.001) compared with NaCl in both participants with type 1 diabetes and control participants. In adipose tissue, 3-OHB reduced protein kinase A phosphorylation of perilipin (*p*<0.001) and hormone-sensitive lipase phosphorylation at Ser660 (*p*<0.001) and Ser563 (*p*<0.01) similarly in participants with type 1 diabetes and control participants. Indices of glucose metabolism remained unaffected throughout in both groups.

**Conclusions/interpretation:**

Our findings indicate that, in individuals with type 1 diabetes, the suppression of lipolysis, blood NEFA concentrations and adipose tissue signalling activity in response to 3-OHB remains intact compared with healthy control participants. These findings imply that derailment of receptor signalling by 3-OHB is unlikely to be involved in the development of diabetic ketoacidosis.

Trial registration: ClinicalTrials.gov NCT04656236

**Funding:**

Open access funding provided by Aarhus Universitet. This study was supported by the Novo Nordisk Foundation (NNF19OC0058872) and the Health Research Foundation of Central Denmark Region.

**Graphical Abstract:**

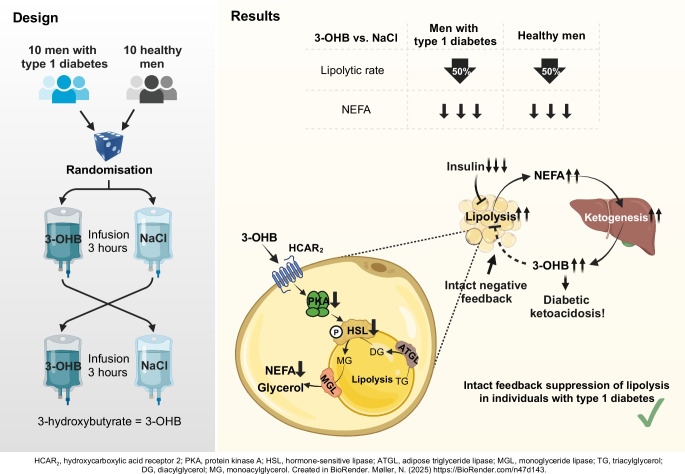

**Supplementary Information:**

The online version of this article (10.1007/s00125-025-06423-5) contains peer-reviewed but unedited supplementary material.


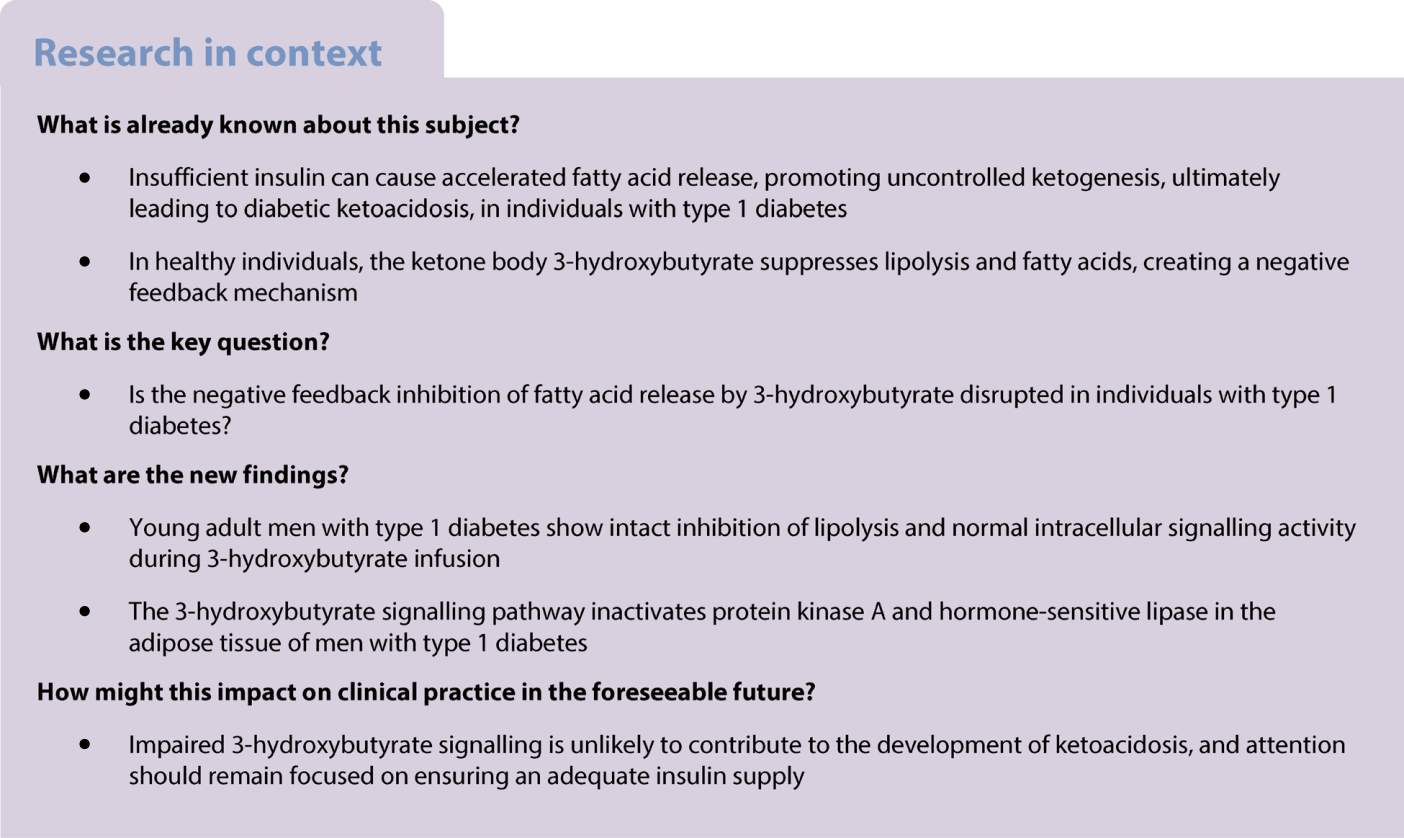


## Introduction

Diabetic ketoacidosis (DKA) is a consequence of absolute insulin deficiency and a potentially life-threatening complication in individuals with type 1 diabetes [1]. The insufficient insulin supply, accompanied by an increased concentration of counter-regulatory hormones, leads to metabolic derangements, including hyperglycaemia, electrolyte disturbances, dehydration and elevated circulating NEFA. The increased NEFA availability and low insulin-to-glucagon ratio favour ketogenesis in the liver and thus ketone body release, ultimately leading to ketoacidosis [2, 3].

Lipolysis is the sequential process of NEFA release from adipose tissue into the bloodstream, regulated by hormonal signals. The most important mediator of lipolytic signalling in adipocytes is the protein kinase A (PKA) pathway (the adenylyl cyclase/cAMP/PKA system), which is predominantly activated by catecholamines. Upon activation, PKA phosphorylates and activates hormone-sensitive lipase (HSL), an essential enzyme that is involved in the breakdown of triacylglycerol to NEFA and glycerol [4]. Insulin serves as a potent inhibitor of lipolysis, and the insulin pathway constitutes the main anti-lipolytic cascade. This pathway involves the phosphorylation and activation of Akt, which subsequently downregulates PKA activity and HSL activation [5]. Notably, the ketone body 3-hydroxybutyrate (3-OHB) inhibits lipolysis by binding to the hydroxycarboxylic acid receptor 2 on the surface of adipocytes, initiating a signalling mechanism that may also involve PKA and HSL inactivation [6, 7]. In healthy individuals, exogenous 3-OHB supplementation effectively suppressed lipolysis and circulating NEFA concentrations, indicating that 3-OHB may serve as a protective negative feedback regulator of lipolysis [6, 8–10]. The primary aim of this study was to investigate the anti-lipolytic effect and associated signalling pathway of 3-OHB in individuals with type 1 diabetes compared with healthy control participants. We hypothesised that the negative feedback suppression of NEFA release by 3-OHB is disrupted in individuals with type 1 diabetes, representing a pathological mechanism that potentially contributes to the development of DKA.

## Methods

### Ethics

The present study was approved by the Central Regional Committee on Health Research Ethics, Denmark (1–10-72–251−20) and was registered at the Danish Data Protection Agency and ClinicalTrials.gov (NCT04656236). Written informed consent was obtained from all participants before participation in the study.

### Participants

**Fig. 1:**
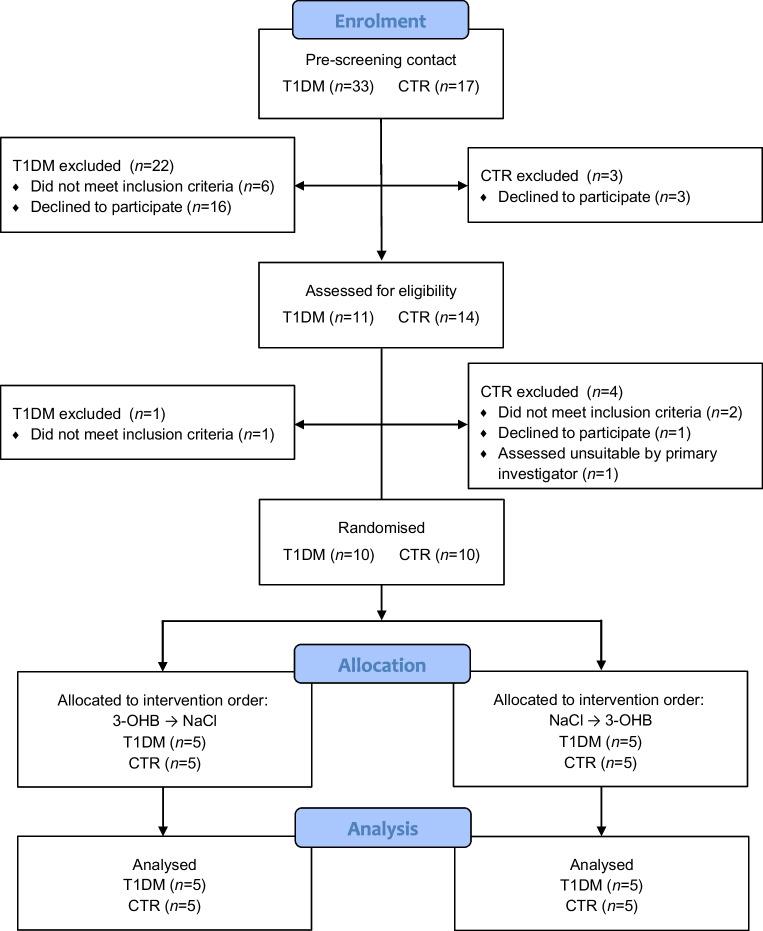
CONSORT flow diagram. T1DM, participants with type 1 diabetes; CTR, healthy control participants

Ten participants diagnosed with type 1 diabetes and ten age-, BMI- and weight-matched control participants from the Central Denmark Region were included in the study (Fig. 1). Participants with type 1 diabetes were recruited between March 2021 and February 2022 from the outpatient clinic at Steno Diabetes Center, Aarhus, and through social media; healthy control participants were recruited only through social media. Inclusion criteria were male sex, age >18 years, and BMI between 19 and 26 kg/m^2^. Individuals with type 1 diabetes were eligible for inclusion if they had a diagnosis of type 1 diabetes and were C-peptide negative (<0.2 nmol/l), while exclusion criteria were severe comorbidity, daily use of medication other than insulin or use of very long-acting insulin analogues (duration of action >24 h). Control participants were healthy male individuals without chronic or acute illness or any regular use of medication. A normal screening examination and blood tests (sodium, potassium, albumin, creatinine, C-reactive protein, leucocytes, alanine aminotransferase, bilirubin, thyroid stimulating hormone, haemoglobin, thrombocytes, international normalised ratio) were required for all participants before they were enrolled in the study. One potential control participant was assessed as unsuitable for participation due to very limited abdominal subcutaneous adipose tissue, making adipose tissue biopsy collection unfeasible. HOMA-IR was calculated for participants as fasting insulin (pmol/l) × fasting glucose (mmol/l)/135. Sex and ethnicity were self-reported. Because of the small sample size of ten participants per group, we chose to include only men, to minimise outcome variation from potential sex differences in metabolism.

### Study design

The study was designed as a randomised, single-blind, crossover trial. All participants were examined on one study day while receiving 3 h i.v. infusions of (i) sodium-d/l-3-OHB (3-OHB, 75 g/l, Sigma-Aldrich, MO, USA; a racemic mixture of d- and l-3-OHB) and (ii) saline (NaCl, 34 g/l; Capital Region Pharmacy, Herlev, Denmark) in random order and separated by a 1 h washout period. The two interventions were matched for sodium content. The 3-OHB infusion was administered as a priming bolus (30 g/h for 15 min), followed by a continuous infusion (10.5 g/h). NaCl was administered in equivalent volumes. A 1 h washout period was considered sufficient to avoid significant carryover, based on the results of previous studies examining the effects of intravenous 3-OHB administration [11].

Five participants were randomised to receive 3-OHB followed by NaCl and five to receive NaCl followed by 3-OHB within both groups. The resource at www.randomizer.org was used for the randomisation process. All participants were blinded to the intervention order. The enrolment of participants, the generation of intervention sequence allocation and the intervention assignment for each participant were conducted by the primary investigator/first author.

### Study day

Examinations were conducted at the Medical/Steno Aarhus Research Laboratory (Aarhus University Hospital, Aarhus, Denmark). All participants were studied after an overnight fast (10 h) and were instructed to avoid strenuous physical activity, alcohol and coffee intake, and to follow the national dietary recommendations (approximately 30% fat, 20% protein and 50% carbohydrate) for 48 h before the study day.

**Fig. 2:**
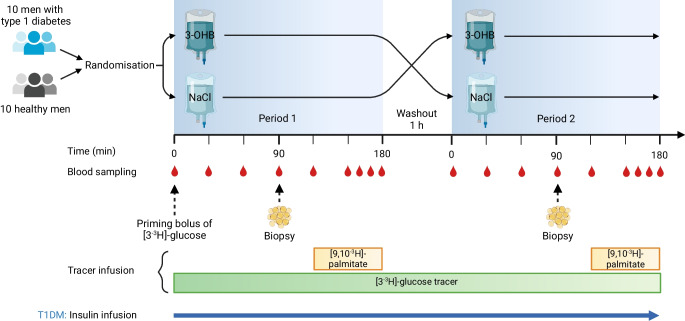
Study flow diagram. Ten men with type 1 diabetes and ten healthy men were examined during 3 h of i.v. infusion with 3-OHB and saline (NaCl) in a randomised crossover design. The timing of blood sampling, adipose tissue biopsy collection and tracer infusions is shown. Participants with type 1 diabetes (T1DM) received a stable insulin infusion throughout the study day. Created in BioRender. Møller, N. (2025) https://BioRender.comd/z88k119

Participants arrived at the research facilities at 07:00 hours and were placed in a bed shortly after. Venous catheters were inserted in a cubital vein for intravenous infusions and in a dorsal hand vein heated by a heating pad for arterialised blood sampling [12]. At approximately 08:00 hours, the first intervention period (3-OHB/NaCl) was initiated; the second intervention period (3-OHB/NaCl) began at approximately 12:00 hours. The timing of examinations, blood sampling and adipose tissue biopsy collection were similar in the two interventional periods, and are schematically shown in the study flow chart (Fig. 2).

### Insulin treatment of participants with type 1 diabetes

**Fig. 3:**
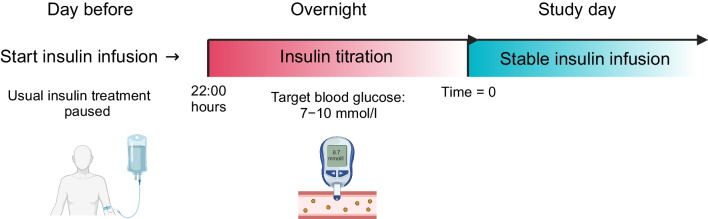
Insulin treatment for participants with type 1 diabetes. Created in BioRender. Møller, N. (2025) https://BioRender.com/i74j440

Long-acting insulin analogue therapy was replaced with subcutaneous rapid-acting insulin 24 h before the study day. Participants with type 1 diabetes were hospitalised at 22:00 hours the evening before the study day to substitute their subcutaneous insulin administration with a continuous intravenous human insulin infusion (Actrapid, Novo Nordisk). Insulin infusion rates were titrated overnight to maintain a blood glucose level of approximately 7–10 mmol/l, as previously described [13]. A glucose sensor was used to measure blood glucose levels once every hour (Freestyle Libre, Abbott, Abbott Park, IL, USA). On the morning of the study day, participants with type 1 diabetes transferred themselves to the research facilities. A continuous insulin infusion rate for the study day was chosen based on the individual’s titrated overnight insulin infusion rate (Fig. 3). The overnight glucose concentrations and insulin infusion rates are provided in the electronic supplementary material (ESM Fig. 1).

### Metabolic fluxes measured using radioactive isotopes

To quantify the lipolytic rate (palmitate flux), a [9,10-^3^H]palmitate tracer was infused (0.666 MBq/h) at time = 120–180 min in each intervention period. Glucose kinetics were estimated by a primed infusion of [3-^3^H]glucose tracer (priming bolus 0.444 MBq, continuous infusion 0.2664 MBq/h) starting at time = 0 min in the first period and continuing throughout the study day. Blood samples were drawn in triplicate during the last 20 min of each intervention period at the time points 160, 170 and 180 min. Plasma palmitate concentration and specific activity were determined by HPLC, while plasma [3-^3^H]glucose specific activity was measured using a liquid scintillation counter. Whole-body palmitate flux (µmol/min) was calculated as palmitate tracer infusion rate (dpm/min) divided by the steady-state specific activity (dpm/μmol) for palmitate as previously described [14]. The glucose rate of appearance (endogenous glucose production, EGP) and rate of disappearance (*R*_d_) were calculated using Steele’s non-steady-state equation [15].

### Adipose tissue biopsies and western blotting

Abdominal subcutaneous adipose tissue biopsies were obtained 90 min after initiating each intervention period by needle aspiration under local anaesthesia (8 ml lidocaine ‘SAD’, 10 mg/ml; Amgros, Copenhagen, Denmark). The biopsies were immediately cleaned for blood contamination and snap-frozen in liquid nitrogen before being stored at −80°C. To extract protein, samples were homogenised at 4°C in PI3 buffer (50 mmol/l HEPES, 137 mmol/l NaCl, 10 mmol/l Na_4_P_2_O_7_, 20 mmol/l NaF, 5 mmol/l EDTA, 1 mmol/l MgCl_2_, 1 mmol/l CaCl_2_, 2 mmol/l Na_3_VO_4_, 5 mmol/l nicotinamide, 1% Halt protease inhibitor cocktail (100×, Thermo Fisher), 1% NP-40, 10% glycerol in demineralised water), and centrifuged for 20 min (16,060 *g*) to separate the protein-containing liquid (infranatant) from the lipid layer and pellet. The protein concentration in each sample was measured using a Pierce BCA protein assay (Thermo Fisher). Laemmli buffer (40 ml glycerol, 8.2 g SDS, 25 ml Tris base/HCl, 0.03 g bromophenol blue, 6.2 g dithiothreitol, 35 ml demineralised water) was added, and protein concentrations were standardised for all samples (1 µg/1 µl) by dilution with demineralised water.

Western blot analyses were performed using the Bio-Rad Criterion system (4–15% Criterion XTBis-Tris gels; Bio-Rad, Hercules, CA, USA) to measure the relative contents of specific protein targets. Gels were placed in running buffer (30.2 g Tris base, 114.2 g glycine, 10 g SDS in 1000 ml demineralised water) during protein loading and the western blot procedure. Membranes with transferred proteins were rinsed and stored in 1× TBS-T buffer (10× includes 2.2 g Tris base, 13 g Tris base/HCl, 87.7 g NaCl, 5 ml Tween-20 in 1000 ml water, adjusted to pH 7.4). The membranes were blocked in 1% BSA in TBS-T before adding the primary antibody. The following primary antibodies were used: hormone-sensitive lipase phosphorylated at Ser563, Ser565 or Ser660 (pHSL) (4139, 4137 and 4126; Cell Signaling Technology, MA, USA), HSL (PA5-17196; Cell Signaling Technology), phospho-PKA substrate (9624; Cell Signaling Technology), perilipin (PLIN1; 9349; Cell Signaling Technology), Akt phosphorylated at Ser473 or Thr308 (4060 and 9275; Cell Signaling Technology), pan Akt (ma5-14916; Thermo Fisher) and the phosphatase and tensin homologue (PTEN; 9188; Cell Signaling Technology). They were diluted in 1% BSA in TBS-T with 0.002% sodium azide. All antibodies were validated prior to use. For the secondary antibody, we used goat anti-rabbit IgG secondary antibody (31460; Thermo Fisher) diluted in 1% BSA in TBS-T. The same western blot membrane was exposed to both phospho-PKA substrate and PLIN1 antibody to identify the band representing PKA phosphorylation of PLIN1 as a measure of PKA activity. Target bands were visualised using the ChemiDoc MP imaging system (Bio-Rad), and quantified using Image Lab 5.0 (Bio-Rad). Total protein normalisation was performed to account for variability in protein loading and transfer using stain-free technology [16]. Western blot data are presented as median ratios of phosphorylated protein/total protein or phosphorylated/non-phosphorylated protein unless otherwise specified, and are plotted as ratio change relative to the median value for control participants during NaCl infusion.

### Blood sample analysis

Blood samples were drawn at baseline and at intervals throughout the study day. Plasma glucose and lactate were immediately measured using immobilised enzyme biosensor technology (YSI 2300 model Stat Plus; Bie & Berntsen, Herlev, Denmark). Blood d-3-OHB was measured using Freestyle Precision ketone test strips (Abbott). All other samples were centrifuged and stored at −20°C for batch analyses after study completion. Commercially available kits were used for quantification of plasma/serum concentrations of insulin (Insulin ELISA; Mercodia, Uppsala, Sweden), C-peptide (C-peptide ELISA; Mercodia), NEFA (FujiFilm/Wako Chemicals Europe, Germany) and glucagon (Glucagon ELISA; Mercodia) in accordance with the manufacturers’ guidelines. Blood total d/l-3-OHB was measured by hydrophilic interaction LC tandem MS [17].

### Endpoints

The primary endpoint of this study was the difference in palmitate flux between groups after 3 h of 3-OHB infusion (time = 180 min). Secondary endpoints were differences in circulating concentrations of NEFA, insulin, glucagon, glucose and lactate, glucose kinetics (time = 180 min), and the relative activity of enzymes involved in lipolytic signalling (time = 90 min).

### Power calculation and statistics

Based on previous work from our laboratory examining the effect of 3-OHB on lipolytic rate in healthy men, a power calculation was performed using α = 0.05, β = 0.8 and an expected reduction in lipolytic rate for participants with type 1 diabetes, which was 30% less than for control participants with an assumed SD of σ = 0.17. This resulted in a necessary sample size of *n*=10 in each group.

All statistical analyses were performed using R software version 4.4.1 (R Foundation for Statistical Computing, Vienna, Austria). Repeated measures were analysed using a mixed model, with time, intervention, group, intervention sequence (order) and period of the day (first or second) as fixed effects, and participants and visit within participants as random effects, followed by pairwise comparisons of estimated means. Baseline data were compared using an unpaired Student’s *t* test and are presented as medians (ranges). Model validation was performed by inspecting QQ plots and histograms of residuals and predicted vs fitted residuals. In the absence of normal distribution, the mixed-model analysis was performed on log-transformed data.

The results were graphically visualised using GraphPad Prism version 10.0.0 for Windows (GraphPad Software, MA, USA). Blood sample and tracer data are presented as curves showing means ± SEM or bar plots showing means ± SD. Western blot results are shown as dot plots with medians. The result estimates in the text are presented as means ± SD. Effect estimates are presented as mean differences or median ratios with 95% CI and *p* value. A *p* value <0.05 was considered statistically significant.

## Results

### Participants

All participants were young adult men (aged 23–39 years) with a BMI of approximately 24 kg/m^2^ (range 21–26 kg/m^2^). Age and anthropometric measures were comparable between groups (Table 1). Participants with type 1 diabetes had a median diabetes duration of 15 years, a median HbA_1c_ of 56 mmol/mol (7.3%), a median serum C-peptide of 0.008 nmol/l and a median HOMA-IR score of 3.5 (Table 1). Participants were ethnically homogeneous and primarily of northern European descent. We believe that the study participants are representative of otherwise healthy, young adult men with type 1 diabetes and healthy young adult men from the Danish population [18]; however, information regarding socioeconomic status was not collected.

**Table 1:** Baseline demographic characteristics

	Controls (*n*=10)	Individuals with type 1 diabetes (*n*=10)	*p* value
Age (years)	27 (23–37)	27.5 (24–39)	0.64
Weight (kg)	78.5 (60–97)	78 (65–109)	0.64
Height (cm)	184 (169–192)	182 (170–204)	0.65
BMI (kg/m^2^)	24.2 (21–26)	24.6 (21–26)	0.74
Creatinine (μmol/l)	68.5 (49–112)	75 (65–88)	0.60
Diabetes duration (years)	–	15 (6–23)	–
HbA_1c_ (mmol/mol)	35 (30–37)	56 (42–78)	<0.001
HbA_1c_ (%)	5.4 (4.9–5.5)	7.3 (6.0–9.3)	
Mean glucose (mmol/l)	5.9 (5.2–6.2)	8.9 (6.9–12.2)	<0.001
C-peptide (nmol/l)	0.73 (0.33–1.89)	0.008 (0.008–0.08)	<0.001
HOMA-IR	0.75 (0.6–1.5)	3.5 (1.7–10.6)	<0.001

Data are shown as median (range) and corresponding *p* value from a two-sample *t* test comparison of healthy control participants and participants with type 1 diabetes

### 3-hydroxybutyrate concentrations

**Fig. 4:**
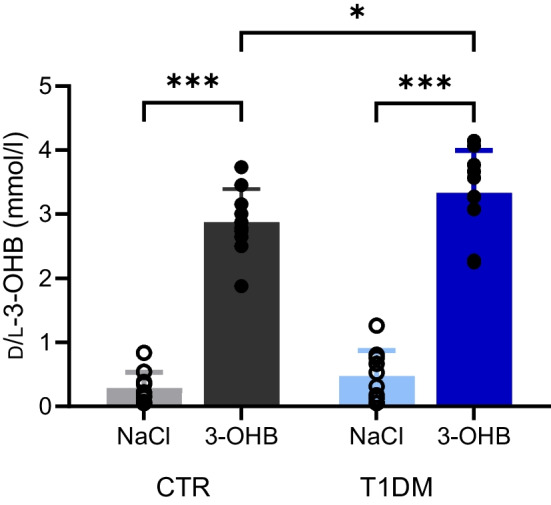
Plasma 3-OHB concentrations. d/l-3-OHB blood concentrations after 3 h of infusion with 3-OHB and saline (NaCl), presented as means ± SD (*n*=10 per group). Data were analysed using a mixed model followed by pairwise comparisons of estimated means: *p<0.05, ***p<0.001. T1DM, participants with type 1 diabetes; CTR, healthy control participants

The blood concentration of total d/l-3-OHB increased to 3.3 ± 0.7 mmol/l in participants with type 1 diabetes and to 2.9 ± 0.5 mmol/l in control participants after 3 h of 3-OHB infusion (mean difference 0.5 mmol/l; 95% CI 0.05, 0.9; *p*=0.03). During NaCl infusion, the mean d/l-3-OHB concentrations remained low at 0.5 ± 0.4 mmol/l in participants with type 1 diabetes and 0.3 ± 0.3 mmol/l in control participants (mean difference 0.2 mmol/l; 95% CI −0.2, 0.6; *p*=0.35) (Fig. 4). Plasma 3-OHB concentrations were generally higher during the second infusion period (period, *p*=0.004), independent of the intervention sequence (order, *p*=0.51). d-3-OHB concentrations during the entire study day are provided in the electronic supplementary material (ESM Fig. 2a).

### Hormone levels

**Fig. 5:**
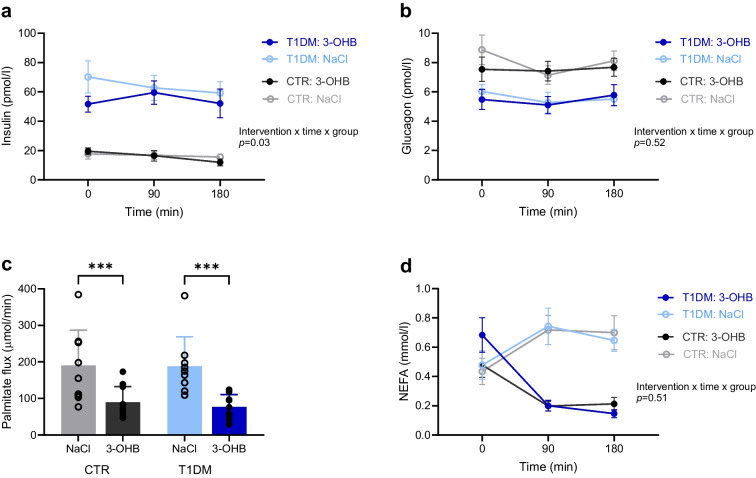
Hormone levels and measures of lipolysis. (**a**, **b**, **d**) Circulating blood concentrations of insulin (**a**), glucagon (**b**) and NEFA (**d**) during the 3 h infusions with 3-OHB and saline (NaCl), presented as means ± SEM (*n*=10 per group). (**c**) Rate of lipolysis (palmitate flux) at 180 min, presented as means ± SD (control participants, *n*=10; participants with type 1 diabetes, *n*=9). Data were analysed using a mixed model followed by pairwise comparisons of estimated means: ***p<0.001. T1DM, participants with type 1 diabetes; CTR, healthy control participants

Peripheral serum insulin concentrations were three- to fourfold higher in participants with type 1 diabetes compared with control participants during both interventions (Fig. 5a). Notably, insulin concentrations were similar when comparing 3-OHB with NaCl within both groups at the time of subcutaneous adipose biopsy collection (time = 90 min) and after 3 h of infusion (time = 180 min). Plasma concentrations of C-peptide were low in participants with type 1 diabetes and stable at around 0.3 nmol/l during both interventions in control participants (ESM Fig. 3). Glucagon concentrations were unaltered throughout the two interventions, with lower concentrations in participants with type 1 diabetes compared with control participants (Fig. 5b). Pairwise comparisons of mean hormone concentrations between groups and interventions are shown in ESM Table 1.

### Lipolytic rate

Infusion of 3-OHB for 3 h suppressed the lipolytic rate (palmitate flux) equally by more than 50% in participants with type 1 diabetes and control participants compared with NaCl (Fig. 5c). In participants with type 1 diabetes, the mean palmitate flux was 78 ± 34 µmol/min during 3-OHB infusion and 181±79 µmol/min during NaCl infusion (mean difference 103 µmol/min; 95% CI 62, 145; *p*<0.001). In control participants, the mean palmitate flux was 90 ± 43 µmol/min during 3-OHB infusion and 190 ± 97 µmol/min during NaCl infusion (mean difference 100 µmol/min; 95% CI 60, 140; *p*<0.001). No differences in mean palmitate flux were observed between participants with type 1 diabetes and control participants following NaCl infusion (mean difference 9 µmol/min; 95% CI −40, 58; *p*=0.71) or 3-OHB infusion (mean difference 12 µmol/min; 95% CI −38, 63; *p*=0.62). The reduced lipolytic rate following 3-OHB infusion was associated with 0.5 mmol/l lower concentrations (95% CI 0.3, 0.7) for NEFA compared with NaCl infusion in both groups (Fig. 5d, *p*<0.001). The lipolytic rates and NEFA concentrations were generally higher in the second intervention period in both groups (period, *p*<0.001; ESM Fig. 2b).

### Lipolysis signalling in adipose tissue

**Fig. 6:**
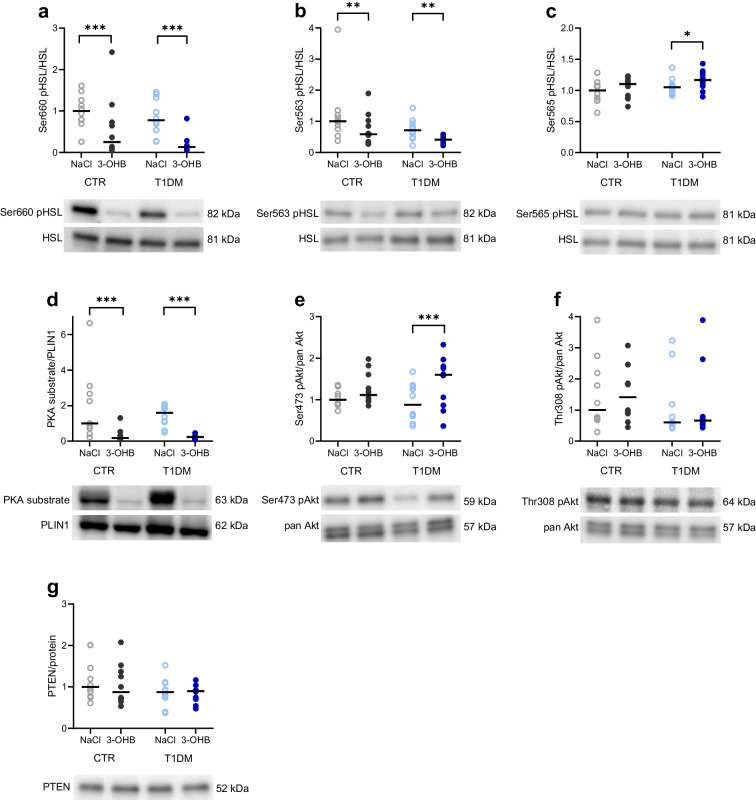
Western blot analyses of subcutaneous abdominal adipose tissue. Relative protein expression during infusions of 3-OHB and saline (NaCl) (*n*=10 per group). (**a**–**c**) HSL phosphorylated at serine 660, 563 or 565 (pHSL) relative to total HSL. (**d**) Phospho-PKA substrate relative to PLIN1 (control participants, *n*=9; participants with type 1 diabetes, *n*=10). (**e**, **f**) Akt phosphorylated at serine 473 or threonine 308 relative to total Akt (pan Akt). (**g**) PTEN relative to total protein. Data are presented relative to the median value for control participants during NaCl infusion. The black horizontal bars indicate the median value, while dots represent each individual. Representative western blots are shown, with the molecular mass (kDa) of the specific bands indicated. A mixed-model analysis with pairwise comparisons was used to compare interventions and groups: *p<0.05, **p<0.01, ***p<0.001. T1DM, participants with type 1 diabetes; CTR, healthy control participants

In subcutaneous abdominal adipose tissue, 3-OHB infusion decreased HSL phosphorylation at Ser660 by approximately 80% in participants with type 1 diabetes (median ratio 0.22; 95% CI 0.13, 0.37; *p*<0.001) and approximately 70% in control participants (median ratio 0.32; 95% CI 0.19, 0.55; *p*<0.001) compared with NaCl (Fig. 6a). Moreover, HSL phosphorylation at Ser563 was reduced by approximately 40% during 3-OHB infusion in both groups compared with NaCl (participants with type 1 diabetes: median ratio 0.6; 95% CI 0.44, 0.82; *p*=0.003; control participants: median ratio 0.61; 95% CI 0.44, 0.84; *p*=0.004) (Fig. 6b). No significant differences between groups were observed, although the phosphorylation levels of HSL were lower in participants with type 1 diabetes in general (Fig. 6a, b). HSL phosphorylation at Ser565 did not differ between interventions in control participants but was slightly increased during 3-OHB infusion in participants with type 1 diabetes (Fig. 6c). PKA phosphorylation of PLIN1 was suppressed by more than 80% during 3-OHB infusion in both groups (participants with type 1 diabetes: median ratio 0.15; 95% CI 0.07, 0.31; *p*<0.001; control participants: median ratio 0.18; 95% CI 0.08, 0.38; *p*<0.001) (Fig. 6d). Additionally, 3-OHB infusion increased Akt phosphorylation at Ser473 in participants with type 1 diabetes (mean difference 0.47; 95% CI 0.23, 0.71; *p*<0.001) compared with NaCl, with only a small, insignificant difference in control participants (mean difference 0.22; 95% CI −0.02, 0.46; *p*=0.08) (Fig. 6e). However, there was no difference when comparing the mean estimates between groups during either 3-OHB or NaCl infusion. Phosphorylation of Akt at the Thr308 site and PTEN were unaffected by intervention or group (Fig. 6f, g).

### Glucose turnover

**Fig. 7:**
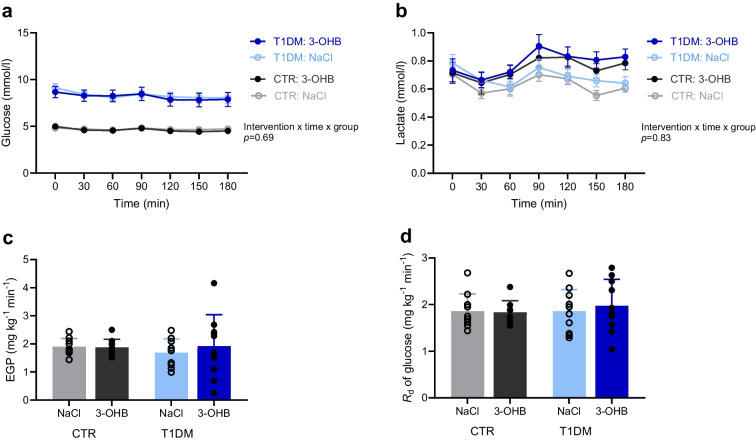
Glucose kinetics and lactate. (**a**, **b**) Circulating blood concentrations of glucose (**a**) and lactate (**b**) during the 3 h infusions with 3-OHB and saline (NaCl), presented as means ± SEM (*n*=10 per group). (**c**, **d**) EGP (**c**) and *R*_d_ (**d**) at time = 160–180 min, presented as means ± SD (*n*=10 per group). Data were analysed using a mixed model followed by pairwise comparisons of estimated means. T1DM, participants with type 1 diabetes; CTR, healthy control participants

The mean plasma glucose concentration during the study day was approximately 8 mmol/l for participants with type 1 diabetes and approximately 5 mmol/l for control participants, and was not influenced by 3-OHB or NaCl infusion in either group (Fig. 7a). During 3-OHB infusion, plasma lactate increased slightly in both groups, reaching a higher concentration after 3 h than during NaCl infusion (mean difference 0.2 mmol/l; 95% CI 0.1, 0.3; *p*=0.001 Fig. 7b). The EGP and *R*_d_ were comparable between interventions and groups (Fig. 7c, d).

## Discussion

The present study was designed to investigate whether the inhibition of lipolysis by 3-OHB is preserved in individuals with type 1 diabetes compared with matched control participants. In young adult men with type 1 diabetes, we showed an intact negative feedback loop whereby increasing circulating 3-OHB concentrations inhibit lipolysis and release of the ketogenic precursor NEFA. However, it is important to note that the current study set-up combines concomitantly elevated 3-OHB and sufficient insulin supply, and cannot necessarily be extrapolated to the stressed metabolic conditions of incipient DKA, which is characterised by absolute insulin deficiency. Moreover, DKA is often accompanied by inflammation [19] and increased levels of counter-regulatory hormones, leading to adipose tissue insulin resistance and, consequently, accelerated lipolysis [20–24]. High glucagon levels are known to stimulate ketogenesis [3], particularly when combined with portal insulinopenia, and the hyperketonaemia may be further aggravated by the reduced ketone body clearance observed during DKA [25]. This pathophysiological cascade may forcefully push the balance towards overall increased lipolysis, and may over-ride the protective anti-lipolytic effect of 3-OHB. Furthermore, the participants with type 1 diabetes were generally well-regulated in terms of their HbA_1c_ levels, and we can only speculate whether the responses may be altered in high-risk individuals with type 1 diabetes and recurrent DKA.

The inhibition of lipolysis by 3-OHB may be facilitated through the hydroxycarboxylic acid receptor 2 [7]. The signalling pathway downstream of hydroxycarboxylic acid receptor 2 involves inactivation of PKA and HSL [6, 26, 27]. This is in line with our findings showing a decline in the phosphorylation and thus inactivation of PKA and HSL during 3-OHB infusion compared with NaCl in both participants with type 1 diabetes and control participants. Insulin is a potent inhibitor of lipolysis, and the anti-lipolytic mechanism of 3-OHB may potentially involve insulin action. In the present study, lipolysis was suppressed by 3-OHB infusion without affecting circulating insulin concentrations. At the signalling level, phosphorylation of the Ser473 residue of Akt was increased by 3-OHB infusion, while phosphorylation of the Thr308 residue remained unchanged. Phosphorylation at the Ser473 site may activate the regulatory domain of Akt, which is believed to stabilise the active conformation of Akt. In contrast, phosphorylation at Thr308 is crucial for activating the Akt kinase domain, mediating downstream Akt activity [28–30]. Therefore, the increased phosphorylation of Akt at Ser473 is unlikely to lead to enhanced downstream Akt activity, as it is not accompanied by an increase in Thr308 phosphorylation. This suggests that the 3-OHB signalling pathway in adipocytes is independent of Akt and insulin action.

Insulin resistance is commonly associated with metabolic syndrome and type 2 diabetes, but is also a significant characteristic in individuals with type 1 diabetes [31]. We found a three- to fourfold higher peripheral insulin concentration in participants with type 1 diabetes than in control participants. Despite these considerably different insulin concentrations, we observed similar lipolytic rates, NEFA concentrations and adipose tissue signalling between the two groups. Additionally, glucose levels were higher in participants with type 1 diabetes, demonstrating that these normal-weight, young individuals with type 1 diabetes were significantly insulin-resistant. We assume that the measured peripheral insulin concentration is a good indication of the insulin availability to skeletal muscles and subcutaneous adipose tissue, although we cannot fully account for the differences between exogenous and endogenous insulin supply, which may lead to varying portal insulin concentrations.

A high adipocyte lipolysis rate and excessive NEFA release have been associated with insulin resistance; therefore, inhibiting lipolysis may improve insulin sensitivity [32, 33]. A recent study demonstrated that 3-OHB infusion suppressed growth hormone-stimulated lipolysis and improved insulin sensitivity during a hyperinsulinaemic–euglycaemic clamp [34]. Furthermore, a cohort study suggested an association between mildly elevated circulating 3-OHB and improved insulin sensitivity [35]. Mechanistically, 3-OHB treatment may enhance adipocyte insulin sensitivity at a post-insulin-receptor level [36], possibly by regulating the transcription factor peroxisome proliferator-activated receptor gamma, which is involved in insulin sensitisation [37]. However, our results suggest that this probably does not include increases in insulin signalling.

A potential overall increased insulin sensitivity should improve glycaemic control. However, we did not find any effect of 3-OHB infusion on plasma glucose or glucose turnover (EGP and *R*_d_). Likewise, a previous study on participants with diabetes failed to show an effect on glucose metabolism [38], while others have demonstrated glucose-lowering effects of 3-OHB infusion [39–41]. The effect may be dose-dependent, requiring higher circulating 3-OHB concentrations than achieved in this study.

We observed slightly higher levels of blood 3-OHB in participants with type 1 diabetes compared with control participants, despite similar infusion rates. This disparity may indicate a higher rate of endogenous ketogenesis or reduced 3-OHB clearance. The NEFA availability to the liver is the rate-limiting factor for 3-OHB production [3], and we found similar concentrations of NEFA between groups. Previous research suggests that clearance of 3-OHB tends to be lower in individuals with type 1 diabetes [39]. The d-3-OHB profile from our study day (ESM Fig. 2a) revealed a potent decrease in d-3-OHB concentrations during washout in participants with type 1 diabetes, although d-3-OHB concentrations did not return to pre-intervention baseline levels. Body composition may also influence 3-OHB concentrations and clearance capacity. A lower lean body mass may explain the higher 3-OHB concentrations in participants with type 1 diabetes. However, we did not measure body composition in this study.

The current study has limitations. Although the crossover design with a 1 h washout period diminishes inter-individual and day-to-day variation, it also introduces a risk of carryover effects on the outcomes. Our statistical analysis indicated that carryover effects were not a significant problem, although the period (first vs second) did affect lipolysis and 3-OHB levels. This is most likely because participants were continuously fasting throughout the study day, together with diurnal variations in metabolism. Importantly, the intervention sequences were evenly distributed within the two groups, and we therefore assume that diurnal differences and extended fasting affected the two interventions to a similar extent. The difference in plasma concentrations of insulin, glucagon and glucose between the two groups may also slightly complicate interpretation of our results. However, these differences reflect the habitual condition in each group, thereby mimicking real-life situations. As only male participants were included, the generalisability of the findings to female individuals with type 1 diabetes may be limited. Additionally, we did not collect information on general activity levels, muscle-to-fat mass or dietary habits, all of which may influence insulin sensitivity [42].

In conclusion, we found intact suppression of lipolysis following 3-OHB infusion in male individuals with type 1 diabetes, indicating that the inhibitory effects of 3-OHB on NEFA release from adipose tissue are preserved. These findings imply that impaired 3-OHB signalling is unlikely to be involved in the initiation of DKA.

## Supplementary Information

Below is the link to the electronic supplementary material.

ESM (PDF 556 KB)

## Data Availability

The corresponding author will make data available upon reasonable request.

## Abbreviations

DKA: Diabetic ketoacidosis
EGP: Endogenous glucose production
HSL: Hormone-sensitive lipase
3-OHB: 3-hydroxybutyrate
PKA: Protein kinase A
PLIN1: Perilipin
PTEN: Phosphatase and tensin homologue
*R*_d_: Rate of disappearance of glucose
