# Supplementary material for: Metabolic effects of 3-hydroxybutyrate infusion in individuals with type 1 diabetes compared with healthy control participants: a randomised crossover trial showing intact feedback suppression of lipolysis
Source: Diabetologia. 2025 Apr 10;68(7):1390–402. doi: 10.1007/s00125-025-06423-5 (PMC12176948; doi:10.1007/s00125-025-06423-5)
Supplement: Supplementary file 1 — ESM (PDF 556 KB) [file 125_2025_6423_MOESM1_ESM.pdf]

Electronic supplementary material (ESM)

	CTR (n=10)			T1DM (n=10)			CTR><T1DM		Mixed model
							NaCl	3-OHB	
	NaCl	3-OHB	Mean diff. [95% CI]	NaCl	3-OHB	Mean diff. [95% CI]	Mean diff. [95% CI]	Mean diff. [95% CI]	
D-3-hydroxybutyrate (mmol/l)	0.3±0.2	1.0±0.3	0.7 [0.5;0.9]	0.5±0.3	1.2±0.4	0.8 [0.6;1.0]	0.2 [-0.05;0.4]	0.3 [0.04;0.5]	Time, $p<0.001$<br>Group, $p=0.79$<br>Intervention x time, $p<0.001$<br>Intervention x group, $p=0.65$<br>Group x time, $p=0.002$<br>Intervention x time x group, $p=0.25$
			$p<0.001$			$p<0.001$	$p=0.12$	$p=0.02$	
D/L-3-hydroxybutyrate (mmol/l)	0.3±0.3	2.9±0.5	2.6 [2.2;3.0]	0.5±0.4	3.3±0.7	2.9 [2.5;3.3]	0.2 [-0.2;0.6]	0.5 [0.05;0.9]	Intervention, $p<0.001$<br>Group, $p=0.02$<br>Intervention x group, $p=0.3$
			$p<0.001$			$p<0.001$	$p=0.35$	$p=0.03$	
Insulin (pmol/l)	16±5	12±8	4 [-4;11]	59±24	52±31	7 [-0.6;15]	44 [25;62]	40 [21;59]	Time, $p=0.13$<br>Group, $p<0.001$<br>Intervention x time, $p=0.59$<br>Intervention x group, $p<0.001$<br>Group x time, $p=0.11$<br>Intervention x time x group, $p=0.03$
			$p=0.34$			$p=0.07$	$p<0.001$	$p<0.001$	
Glucagon (pmol/l)	8±2	8±2	0.4 [-0.8;1.7]	6±1	6±2	0.2 [-1;1.4]	3 [0.7;5]	2 [0.04;4]	Time, $p=0.9$<br>Group, $p=0.03$<br>Intervention x time, $p=0.15$<br>Intervention x group, $p=0.38$<br>Group x time, $p=0.91$<br>Intervention x time x group, $p=0.52$
			$p=0.48$			$p=0.75$	$p=0.009$	$p=0.05$	
NEFA (mmol/l)	0.7±0.4	0.2±0.1	0.5 [0.3;0.7]	0.6±0.2	0.1±0.1	0.5 [0.3;0.7]	0.05 [-0.2;0.3]	0.07 [-0.2;0.3]	Time, $p=0.003$<br>Group, $p=0.07$<br>Intervention x time, $p<0.001$<br>Intervention x group, $p=0.24$<br>Group x time, $p=0.1$<br>Intervention x time x group, $p=0.51$
			$p<0.001$			$p<0.001$	$p=0.64$	$p=0.55$	
Glucose (mmol/l)	4.7±0.4	4.5±0.3	0.2 [-0.6;1.0]	8.1±1.8	7.9±2.4	0.2 [-0.7;1.0]	3.3 [2.2;4.5]	3.4 [2.2;4.5]	Time, $p=0.21$<br>Group, $p<0.001$<br>Intervention x time, $p=0.93$<br>Intervention x group, $p=0.27$<br>Group x time, $p=0.79$<br>Intervention x time x group, $p=0.69$
			$p=0.61$			$p=0.67$	$p<0.001$	$p<0.001$	
Lactate (mmol/l)	0.6±0.1	0.8±0.2	0.2 [0.1;0.3]	0.6±0.1	0.8±0.2	0.2 [0.1;0.3]	0.04 [-0.1;0.2]	0.04 [-0.1;0.2]	Time, $p<0.001$<br>Group, $p=0.81$<br>Intervention x time, $p=0.03$<br>Intervention x group, $p=0.39$<br>Group x time, $p=0.73$<br>Intervention x time x group, $p=0.83$
			$p=0.001$			$p<0.001$	$p=0.66$	$p=0.58$	

Table 1: Hormone and substrate concentrations. Data are shown as means±SD (*n*=10 per group) after 3 h of intervention (time=180 min), and mean difference [95% CI] with corresponding *p*-value from pairwise comparisons within each group and between groups during the two interventions. Data were analysed using a mixed model followed by pairwise comparisons of estimated means. T1DM, participants with type 1 diabetes; CTR, healthy control participants

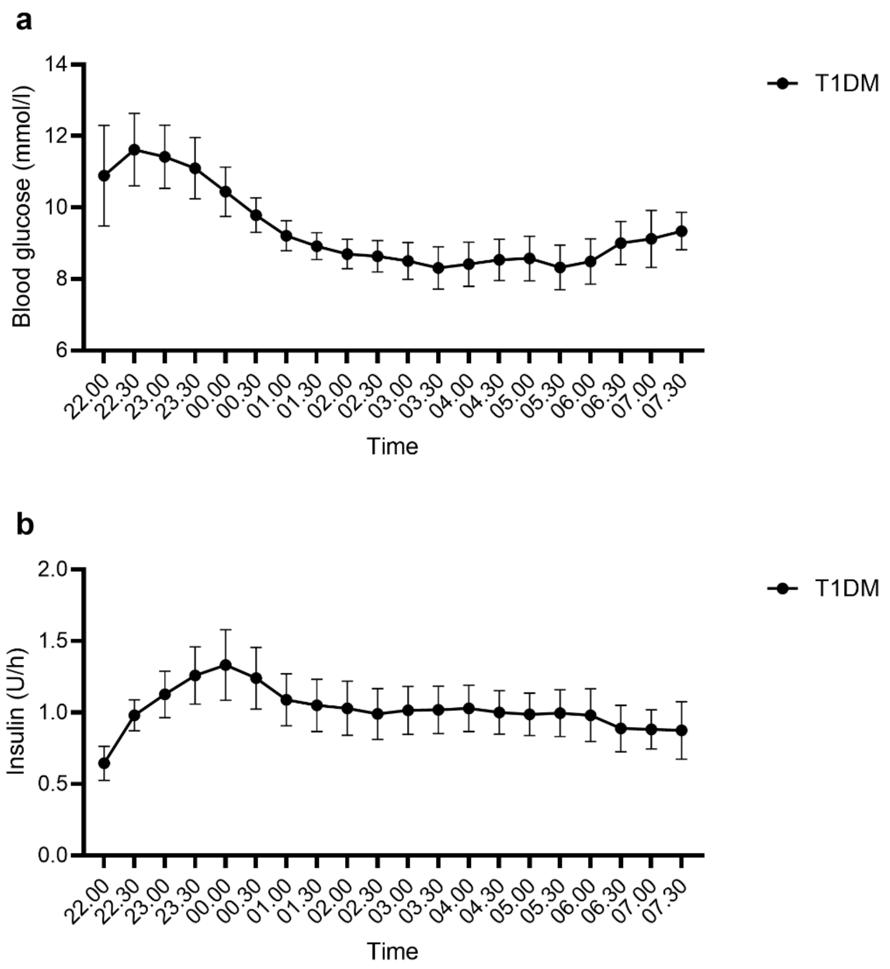

Figure 1: Blood glucose concentrations (a) and insulin infusion rate (b) during the insulin titration overnight before the study day in participants with type 1 diabetes (T1DM). Data are shown as means±SEM (*n*=10).

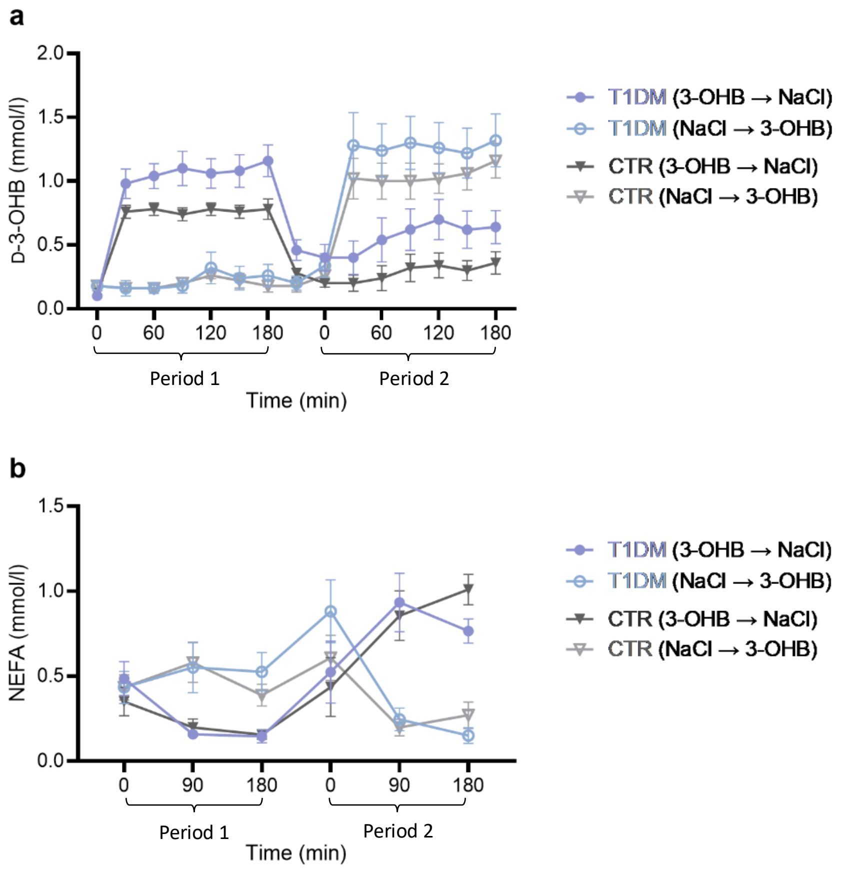

Figure 2: D-3-OHB (a) and NEFA (b) concentrations during both intervention sequences (3-OHB → NaCl and NaCl → 3-OHB). Data are shown as means±SEM (*n*=5 per group). T1DM, participants with type 1 diabetes; CTR, healthy control participants.

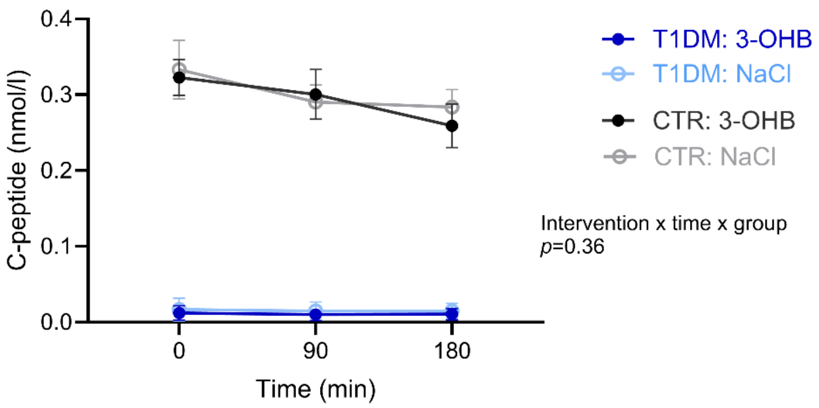

Figure 3: Serum C-peptide concentrations during the 3 h infusions with 3-OHB and NaCl. Data are shown as means±SEM (*n*=10 per group). Data were analysed using a mixed model followed by pairwise comparisons of estimated means. T1DM, participants with type 1 diabetes; CTR, healthy control participants.
